# A Facile Approach to the Hydrothermal Synthesis of Silica Nanoparticle/Carbon Nanostructure Luminescent Composites

**DOI:** 10.3390/ma15238469

**Published:** 2022-11-28

**Authors:** Yuliya A. Podkolodnaya, Alina A. Kokorina, Irina Yu. Goryacheva

**Affiliations:** Department of Inorganic Chemistry, Chemical Institute, Saratov State University, Astrakhanskaya Street 83, 410012 Saratov, Russia

**Keywords:** carbon nanostructures, silica nanoparticles, composite nanoparticles, hydrothermal synthesis, luminescence

## Abstract

Luminescent carbon nanostructures (CNSs) have been intensively researched, but there is still no consensus on a fundamental understanding of their structure and properties that limits their potential applications. In this study, we developed a facile approach to the synthesis of luminescent composite SiO_2_ nanoparticles/CNSs by the targeted formation of a molecular fluorophore, as the significant luminescent component of CNSs, on the surface of a silica matrix during a one-stage hydrothermal synthesis. Silica nanoparticles were synthesized by reverse microemulsion and used as a matrix for luminescent composites. The as-prepared silica nanoparticles had a functional surface, a spherical shape, and a narrow size distribution of about 29 nm. One-stage hydrothermal treatment of citric acid and modified silica nanoparticles made it possible to directly form the luminescent composite. The optical properties of composites could be easily controlled by changing the hydrothermal reaction time and temperature. Thus, we successfully synthesized luminescent composites with an emission maximum of 450 nm, a quantum yield (QY) of 65 ± 4%, and an average size of ~26 nm. The synthesis of fluorophore doped composite, in contrast to CNSs, makes it possible to control the shape, size, and surface functionality of particles and allows for avoiding difficult and time-consuming fractionation steps.

## 1. Introduction

Luminescent CNSs are broad family of nanomaterials with a range of structures [1,2]. Their structure can be graphitic [3], amorphous [4] or include a C_3_N_4_ crystalline core [5]. Typically, CNSs have small size (≤10 nm) and a functionalized surface [6]. They demonstrate fascinating bright and tunable emission from ultraviolet to infrared, high photostability, low toxicity, and excellent solubility in both polar and nonpolar solvents and biocompatibility, as well as wide range of synthetic ways and starting compounds [1,2,6].

CNSs can be synthesized by top-down and bottom-up approaches [7]. Top-down approaches refer to the physical or chemical decomposition of larger carbon materials such as graphite [8], carbon nanotubes [8], graphite oxide [9], graphene oxide (graphene oxide is chemically similar to graphite oxide, but structurally it is very different) [10,11,12], activated carbon [13], nanofibers [14], and soot [15] into smaller carbon fragments, including CNSs. The widespread top-down approaches are arc discharge [16], lasers [17], and electrochemical methods [18]. These methods are usually multistage with labor-intensive processes. Their main disadvantages are large size distributions of CNSs, low QYs, and limited surface functionality [7,19].

Bottom-up methods involve the dehydration, condensation, and carbonization of various starting compounds for the formation of CNSs [7,19,20]. The most popular and efficient methods for large-scale synthesis are solvothermal synthesis [21], microwave synthesis [22], and pyrolysis [23]. In this case, various natural substances (Bombyx mori silk [24], eggshell membrane [25], chitosan [26], etc.) or standardized chemical compounds (citric acid (CA) [20,27,28], folic acid [29], ascorbic acid [30], etc.) can be used as carbon sources. CA is one of the most common reagents for the synthesis of CNSs by bottom-up methods. CA contains three carboxyl groups and a hydroxyl group, which allow for forming noncovalent interactions (hydrogen bonds) and react with other organic compounds [20,27,28]. Doping CNSs produced from CA with nitrogen atoms greatly increases the emission intensity [20]. Therefore, co-reagents such as 1,2-ethylenediamine (EDA) [20,31,32], hexamethylenetetramine [32], triethanolamine [32], ammonia [33], and urea [33,34] are usually added to the synthesis.

The synthetic products prepared from CA and amine-containing agents by bottom-up approaches were investigated [35,36]. A range of approaches were commonly used to purify and separate the obtained products, such as ultracentrifugation with a hydrophilicity gradient [37], liquid chromatography [38], gel electrophoresis [39], dialysis [20], and column chromatography [20]. A detailed exploration of the obtained products allowed for determining the presence of polymer structures, carbon cores, and the molecular fluorophore 1,2,3,5-tetrahydro-5-oxoimidazo [1,2-a]pyridine-7-carboxylic acid (IPCA) [20,35,36,37,38,39]. The obtained fluorophore can be in an unbound state or be associated with the carbon core or polymer structures by noncovalent/covalent bonds [20,35,36,37,38,39]. It has been clearly shown that IPCA makes the main contribution to the photoluminescence of CNSs obtained from CA and amine-containing agents. The main problem with this type of CNS is the lack of reaction control at high heat and pressure. This fact limits the exploration of their structural features, emission mechanism, and the relationship between the structure and the photophysical behavior of CNSs, as well as obtaining a homogeneous product without stages of purification and separation. Recent research demonstrates that the optical properties of CNS are typically presented by IPCA-like organic fluorophores. Thus, there is a uniform terminology for the IPCA-like fluorophores, graphitic, and amorphous nanoparticles as CNSs [40].

At the same time, the terminology for this type of luminescent carbon nanomaterial has not yet become generally accepted. Thus, the observed nanoobjects are also called carbon dots [17,18,19,20,21,22,23,24,25,26,27,28,29,30,31,32], C-dots [36], carbon particles [38], carbon quantum dots [41], etc.

Taking into account the listed problems, we reported a novel approach for the obtainment of composite nanoparticles by the single-stage targeted synthesis of a highly luminescent fluorophore on the surface of the silica matrix; we know that the reported approach was used for the first time. The inclusion of CNSs into silica particles or their grafting onto the silica surface is ordinarily used for the synthesis of composites of similar types [42]. We synthesized silica nanoparticles with surface carboxylic groups, which provided colloidal stability, and amino groups, which are necessary for the formation of a luminescent molecule. To form luminescent structures, the resulting silica nanoparticles were hydrothermally treated with CA. The replacement of the unknown carbon structure binding the fluorophore with the silica nanoparticles makes it possible to control the size and shape as well as the surface functionality of the resulting composites. In addition, we studied in detail the conditions for the synthesis of the matrix and the formation of an IPCA type fluorophore on the matrix surface to obtain stable highly luminescent nanoparticles.

## 2. Materials and Methods

### 2.1. Materials and Apparatus

CA monohydrate (≥99.0%), polyethylene glycol dodecyl ether (Brij L4), tetraethyl orthosilicate (TEOS) (≥98.0%), ammonia solution 28–30% (≥99.0%), N^1^-(3-Trimethoxysilylpropyl) diethylenetriamine (DETAS), quinine hemisulfate salt monohydrate, and ethanol absolute (≥99.8%) were purchased from Sigma-Aldrich (Hoeilaart, Belgium). Hexane (≥99.0%) was purchased from Chimmed (Russia). Carboxyethylsilanetriol sodium salt (CEST) 25% in water was purchased from ABCR (Germany). The used water for the experiments was double-distilled.

Stainless steel autoclaves, ultrasonic bath, centrifuge, and dialysis bags (MWCO = 1000 Da) were used for the synthesis and purification of composite nanoparticles.

### 2.2. Structural Characterization

The structural morphology and size of obtained nanoparticles was observed by transmission electron microscope (TEM) Libra 120 (Carl Zeiss, Oberkochen, Germany). The size processing of the obtained TEM images was carried out with ImageJ software and analyzed using mathematical statistics. The average diameter, polydispersity, and zeta-potential (ζ-potential) of synthesized nanoparticles were analyzed with a Zetasizer Ultra (Malvern Panalytical, Worcestershire, UK). UV/Vis spectra were recorded by Shimadzu UV-1800 (Shimadzu Corporation, Kyoto, Japan). The luminescent spectra were obtained by Cary Eclipse spectrometer (Agilent Technologies, Mulgrave, Victoria, Australia). FTIR-spectra were obtained with an IRAFFINITY-1 FTIR spectrometer in KBr pellets (Shimadzu Corporation, Kyoto, Japan). ^1^H-NMR spectra were recorded at 20–25 °C on a Varian-400 spectrometer (400 MHz; Agilent Technologies, Santa Clara, CA, USA), using D_2_O as a solvent and 4,4-dimethyl-4-silapentane-1-sulfonic acid as an internal standard. DRON-8T X-ray diffractometer was applied for X-ray diffraction studies (XRD) (Bourevestnik, Saint Petersburg, Russia). The powder was poured onto a Si low-background substrate. To register diffraction patterns, we used CuKα radiation, a Goebel mirror (AXO Dresden GmbH, Dresden, Germany), Mythen 2R1D strip detector (640 strips, Dectris, Baden-Daettwil, Switzerland) with 2θ increment of 0.0144°. Pattern recorded in the 2θ range from 10 to 60° by points with a step of 0.2° for the central detector strip; exposure time of 12 s at point.

### 2.3. Fabrication of Silica Nanoparticles Modified with Carboxylic and Amino Groups

Silica nanoparticles were obtained through reverse microemulsion. The microemulsion solution was prepared by mixing 4000 μL of hexane, 1280 μL of surfactant Brij L4, and 240 μL of aqueous ammonia (3%). The ammonia solution acts as a catalyst for the hydrolysis of TEOS. The microemulsion solution was stirred for 1 h. After that, 60 μL of TEOS was added, and the reaction mixture was stirred for 24 h. The modification steps included the addition of 3 μL of 10-times diluted DETAS. Finally, after 24 h, 3 μL of CEST was added and stirred again for 24 h. The synthesized nanoparticles were purified by adding 1500 μL of ethanol to the reaction mixture and centrifuged at 3500× *g* for 7 min. After removal of the supernatant, the sediment silica nanoparticles were washed twice with hexane, ethanol, and water. At each stage of the purification, the reaction mixture was processed in an ultrasonic bath for 1 h.

### 2.4. Hydrothermal Synthesis of Luminescent Composite Nanoparticles

Equal volumes of 1 mL water solutions of CA (0.01 M) and silica nanoparticles were mixed and sonicated for 2 min. The obtained reaction mixture was transferred to the stainless-steel autoclave and heated for 1.5/3 h at 140/160/180/200 °C. After the reaction, the autoclave was cooled at 25 °C. Synthesized composite nanoparticles were purified by centrifugation at 75× *g* for 2 min. The supernatant containing luminescent composites was collected and stored at 4 °C with no exposure to light. The lyophilized product yield was from 4 to 8 mg after the synthesis.

### 2.5. Dialysis of Luminescent Composite Nanoparticles

Luminescent composite nanoparticles were purified by dialysis. The dialysis process was performed using dialysis bag (MWCO = 1000 Da) containing 2 mL of sample in 2 L of double-distilled water for 6 h. Double-distilled water was changed every two hours.

### 2.6. QY Measurement

The QY of the obtained luminescent composite nanoparticles was measured with standard dye. In this method, quinine sulfate was used as a standard dye. It was diluted by 0.1 M H_2_SO_4_ solution, and the QY was 54%. To reduce the reabsorption effect, all samples were diluted to absorbance of 0.1. The QY of the samples was calculated with the following equation:

(1) $${\mathrm{QY}}_{x}={\mathrm{QY}}_{\mathrm{st}}\times \left(\frac{I_{x}}{I_{\mathrm{st}}}\right)\times \left(\frac{A_{\mathrm{st}}}{A_{x}}\right)\times \left(\frac{\eta_{x}^{2}}{\eta_{\mathrm{st}}^{2}}\right)$$

The subscript “x” represents composite nanoparticles and the subscript “st” represents standard of quinine sulfate. The letters “I”, “A” and “η” mean integrated fluorescent emission intensity, refractive index of the solvent, and absorbance, respectively. The excitation and emission wavelengths of QY measurement were 350 and 450 nm, respectively.

## 3. Results and Discussion

To synthesize a luminescent nanocomposite with a specified structure, we applied a new approach for a targeted synthesis of a molecular fluorophore on the surface of a silica matrix with defined morphological properties (size and shape). Silica nanoparticles are one of the universal matrices due to their optical transparency, variety of surface modification reagents for further functionalization, biocompatibility, low toxicity, and high colloidal stability [42,43,44,45,46,47].

Silica nanoparticles can be synthesized by the traditional Stober method [42,45], inverse microemulsion [45,48,49,50,51,52,53], or sol–gel [54,55]. In this work, we used the reverse microemulsion method to synthesize silica nanoparticles with the ability to control their size and shape.

This method involves the formation of reverse micelles, which serve as microreactors for the hydrolysis and condensation of silanes. Due to the similar sizes of micelles, silica nanoparticles have a narrow size and shape distribution. Since the morphology of the obtained silica nanoparticles depends on the shape and size of microdroplets, it is important to determine the composition and molar ratio of the three-component system: organic solvent–surfactant–water. Moreover, the determination of ammonium hydroxide concentration, synthetic time, the amount of TEOS, and/or other organosilanes to modify the surface of nanoparticles is necessary [42,44,45,48].

**Figure 1:**
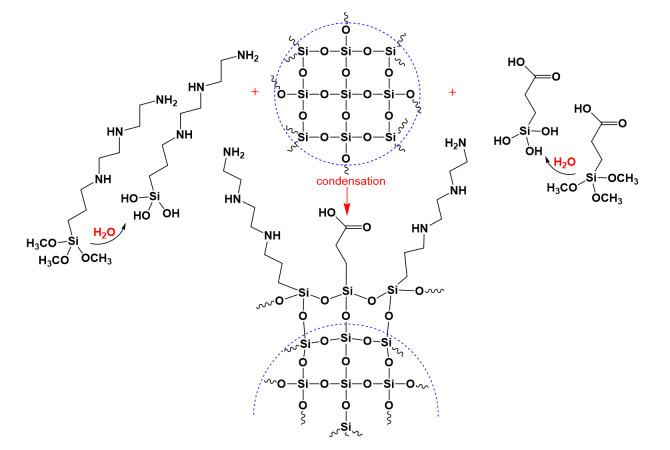
Schematic illustration of silica particles modified with DETAS (**left**) and CEST (**right**).

The reverse microemulsion can be obtained by various combinations of nonpolar phases, water, and surfactants. The most popular surfactants according to the authors [49] are ionic bis (2-ethylhexyl) sulfosuccinate sodium salt [50] and cetriltrimethylammonium bromide [51] and nonionic: Triton X-100 [48], Igepal CO-520 [52] and Brij L30/Brij L4 [49,53] with cyclohexane, n-hexane, and n-heptane as the corresponding nonpolar phase. In this work, to form a reverse microemulsion, we used the nonpolar solvent hexane as an organic phase and the nonionic surfactant Brij L4. According to Goftman et al. [53], the optimal molar ratio in the three-component system hexane–Brij L4–water for the formation of a microemulsion is 3.75:0.4:1. At the stage of micelle formation, we used a 3% aqueous ammonia, since it acts as a catalyst for the following hydrolysis of TEOS inside the micelles. During the hydrolysis, TEOS was transformed into orthosilicic acid. Within stirring for 24 h, the orthosilicic acid entered into polycondensation reactions with the formation of silica nanoparticles limited by the size of micelles. The hydrodynamic size of the obtained particles was 62 ± 1 nm with the polydispersity index (PI) of 0.16 ± 0.04. Synthesized by hydrolysis and condensation of TEOS silica nanoparticles had surface hydroxyl groups. For the following modifications, surface hydroxyl groups interacted with DETAS and CEST according to the scheme in Figure 1. The amount of the modifiers should be less than 1% of the TEOS amount to avoid the aggregation process.

The modification of silica nanoparticles only by DETAS led to the part-compensation of their negative surface charge. As a result, colloidal stability reduced, and a strong aggregation of particles in the water solution occurred (Table 1). Therefore, it was necessary to introduce negatively charged organosilane containing carboxyl, phosphonate, or other groups. In this work, CEST was used as a stabilizing silane. The colloidal stability and morphological characteristics of silica nanoparticles with different percentages of DETAS and CEST (ratios from 50:50 to 0:100, respectively) for the surface functionalization were evaluated using dynamic light scattering (Table 1). A CEST of less than 50% of the total surface functional led to low colloidal stability and the aggregation of silica nanoparticles. An increase in CEST to 50–100% led to a significant decrease in the PI and the hydrodynamic size from 165 to 66 nm. Moreover, a high number of carboxyl groups on the silica surface allowed for increasing the negative ζ-potential as well as their colloidal stability. To obtain high-luminescence and colloid-stable composites, silica nanoparticles with DETAS:CEST ratios of 50:50, 40:60, and 20:80 were hydrothermally treated with CA.

Several research groups reported [56,57] about the stress luminescence of silica materials. It is the light emission process under the applied force. However, the authors pointed out a lack of understanding of the structure–stress–luminescence relationships. Moreover, stress luminescence is not repeatable because the materials are damaged after emitting. The composites developed in this work did not exhibit stress luminescence.

**Figure 2:**
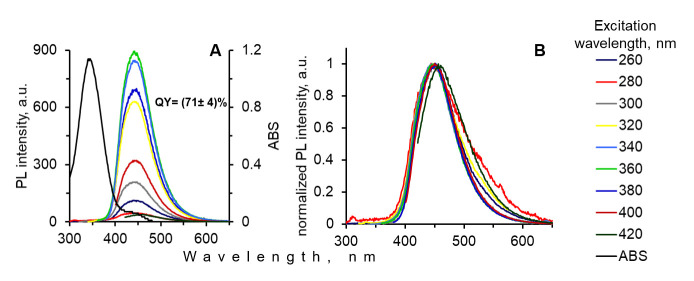
Absorbance and luminescence spectra (**A**), and normalized luminescence spectra (**B**) of IPCA fluorophore purified by dialysis.

According to Song [20], the hydrothermal treatment of CA and EDA leads to a double cyclization reaction between primary amino groups of EDA and two carboxylic groups of CA with the forming of IPCA organic fluorophore (Figure S1). The structure of IPCA was proven with the ^1^H-NMR spectrum (Figure S2). IPCA can be in a free molecular form or a bound form with the products of the interaction of CA and EDA. Moreover, IPCA was responsible for the bright luminescence of CNSs obtained from CA and EDA. The emission of IPCA is in the area of 450 nm (Figure 2A,B).

**Figure 3:**
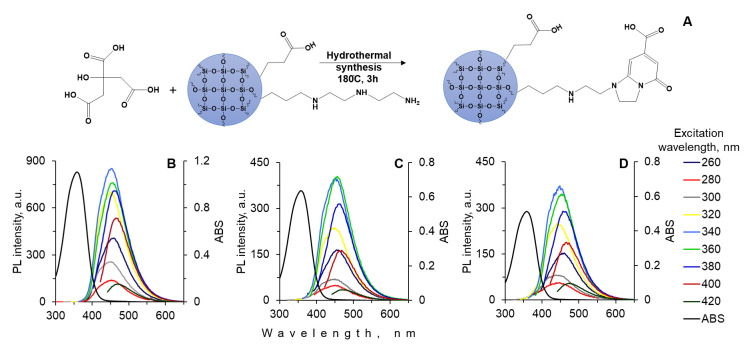
Schematic illustration of the IPCA-type fluorophore formation on the surface of a silica matrix (**A**); the absorption and luminescence spectra of composite nanoparticles with different ratios of the silica surface modifiers of DETAS and CEST: 50:50 (**B**), 40:60 (**C**), and 20:80 (**D**) at different excitation wavelengths.

In this work, an IPCA-type molecular fluorophore was formed directly on a silica matrix as a result of the interaction of CA and silica amino groups (Figure 3A), similar to the mechanism of fluorophore formation from CA and EDA. The synthesized composites of CA and silica matrix with optimal ratios of DETAS and CEST had similar optical profiles. The absorption maximum was centered at approximately 360 nm, which corresponded to the π→π^∗^ or *n*→π^∗^ transitions. The intense blue luminescence at 450 nm was observed at excitation wavelengths of 340–360 nm. After increasing the excitation wavelengths, the emission shifted from 440 to 480 nm with remarkable decreases in luminescent intensity. The low-intensity luminescent red shift is the consequence of luminescent side-products that are formed from CA during the hydrothermal synthesis [20].

The luminescence intensity of the obtained composites depended on the number of amino groups contained on the surface of the silica matrix. Thus, the matrix contained the highest number of amino groups on the surface (the ratio of DETAS:CEST = 50:50) allowed for obtaining composites with the highest luminescence intensity (Figure 3B). The QY of these composites was 66 ± 2%. The composites with a silica matrix of 20:80 DETAS:CEST ratio had the lowest luminescence intensity and QY of 37 ± 3% (Figure 3D). The silica matrix with a DETAS:CEST ratio of 40:60 allowed for obtaining of the composite with intermediate luminescence and QY of 43 ± 4% (Figure 3C).

Dynamic light scattering data were obtained to characterize the size, dispersion, and charge of the prepared composite nanoparticles (Table 2). Composites obtained from silica nanoparticles containing DETAS and CEST on the surface in a ratio of 20:80 were characterized by the highest PI 0.56 ± 0.04. This indicated a high scatter in the sizes of the composite particles and obtaining a highly polydisperse product. Composites obtained from CA and a silica matrix with a 50:50 DETAS:CEST ratio had the highest colloidal stability (ζ-potential about −31 mV). Moreover, these composites had low dispersity (PI = 0.27 ± 0.04) and the highest luminescent QY 66 ± 2%. The silica matrix of 40:60 DETAS:CEST ratio led to the synthesis of composites with intermediate hydrodynamic size, PI. ζ-potential, and QY. However, the ζ-potential of the obtained composites decreased in comparison with the silica matrix. This fact correlates with possible interaction during hydrothermal treatment between carboxyl and amino groups on the surface of the matrix. These interactions can lead to the compensation of the surface negative charge and reduce the reactions between CA and amino groups for IPCA formation.

**Figure 4:**
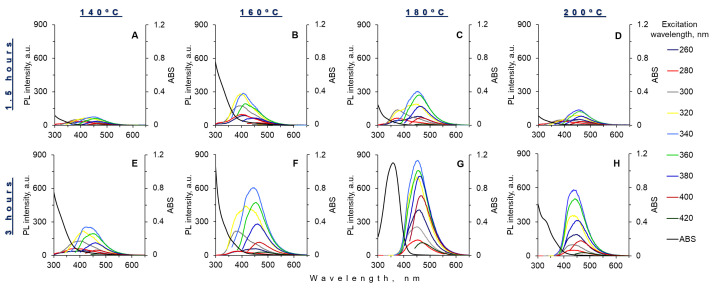
Absorbance and luminescence spectra of composite nanoparticles obtained by hydrothermal synthesis at 140 °C (**A**,**E**), 160 °C (**B**,**F**), 180 °C (**C**,**G**) and 200 °C (**D**,**H**) for 1.5 h and 3 h, respectively.

In order to find the optimal synthetic conditions for obtaining high emissive and colloidal stable composites, we varied the time and temperature of hydrothermal treatment. Hydrothermal heating at 140, 160, 180, and 200 °C for 1.5 and 3 h was used (Figure 4). These synthetic conditions are widespread for the IPCA fluorophore formation. Composites synthesized at 180 °C for 3 h had the highest luminescence intensity (Figure 4G). The optical characteristics of obtained composites at optimal conditions correlated with the spectral characteristics of the IPCA molecular fluorophore. This may be evidence of the formation of an IPCA-type fluorophore on the surface of the silica matrix under hydrothermal treatment.

**Figure 5:**
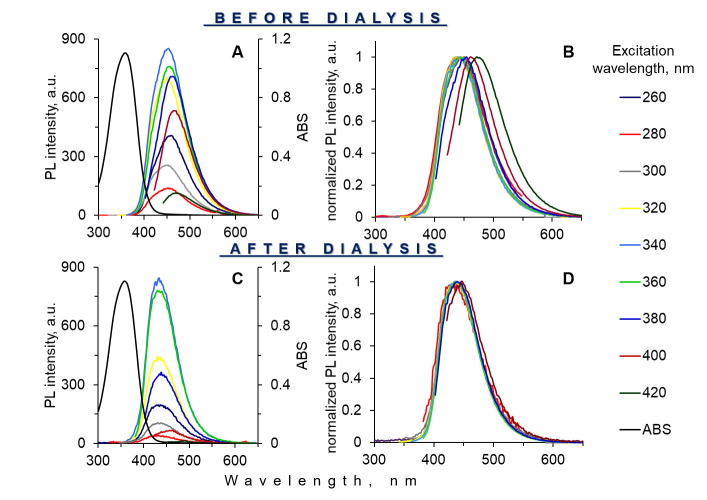
Absorption (black) and luminescence spectra (**A**,**C**), normalized luminescence spectra (**B**,**D**) of composite nanoparticles before and after dialysis, respectively.

The dependence of the emission bands on excitation in the composite nanoparticles may indicate the presence of other emission centers formed by the condensation of CA. Dialysis was used to purify luminescent composites from these emitters, as well as unreacted components, and prove the formation of a fluorophore on the matrix surface. In the dialysis process, the small compounds (CA and/or the products of their condensation) can penetrate the membrane (dialysate). By contrast, the large compounds (composites) cannot pass through the membrane (retentate). Figure 5 illustrates absorption and luminescent spectra before (Figure 5A,B) after dialysis (Figure 5C,D). The absorption and luminescent maxima were retained at 360 and 450 nm, respectively. Moreover, the dependence of the luminescent maximum shift on the excitation wavelengths was not observed (Figure 5D). This indicates that the IPCA-like fluorophore formed and fixed on the silica matrix surface. Moreover, this fact was proven by ^1^H-NMR data containing the signals of the IPCA molecule and protons of the methylene units of the modifiers of a silica matrix (Figure S3A,B). The XRD pattern of composite nanoparticles displayed a broad band centered at 2θ = 22° suggesting the formation of silica nanoparticles modified with IPCA (Figure S4). The pattern also confirmed the presence of the amorphous nature of the silica nanoparticles with an absence of a crystalline phase [58]. The QY of the luminescent composite after dialysis was 65 ± 4%. The QY of the obtained composites is comparable to the QY of silanized semiconductor quantum dots [59] and organic dyes encapsulated in silica [60].

**Figure 6:**
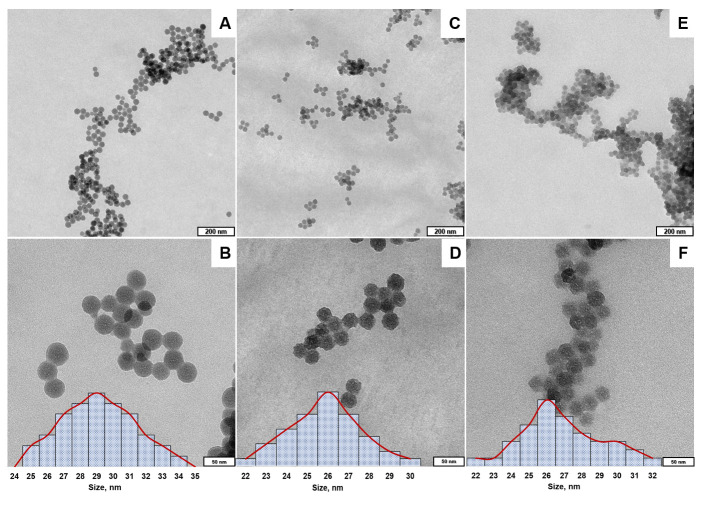
TEM images of silica nanoparticles with amino- and carboxymodifiers (**A**,**B**), luminescent composite nanoparticles before (**C**,**D**) and after dialysis (**E**,**F**).

TEM images of the obtained particles are shown in Figure 6 and Figure S5. They demonstrate lyophilized silica nanoparticles with amino- and carboxymodifiers (Figure 6A,B) and luminescent composite nanoparticles before (Figure 6C,D) and after dialysis (Figure 6E,F). The nanoparticles exhibited good dispersibility and spherical form. Silica nanoparticles modified with carboxyl and amino groups had an average size of 29 ± 5 nm. After hydrothermal treatment, the average size of all composites was reduced compared with the initial silica nanoparticles by ~3 nm and amounted to 26 ± 4 nm. This fact can be explained by the vitrification of silica nanoparticles at high temperatures and pressure during the hydrothermal synthesis. The morphological characteristics of the composite nanoparticles did not change after dialysis; their average size was 26 ± 5 nm.

**Figure 7:**
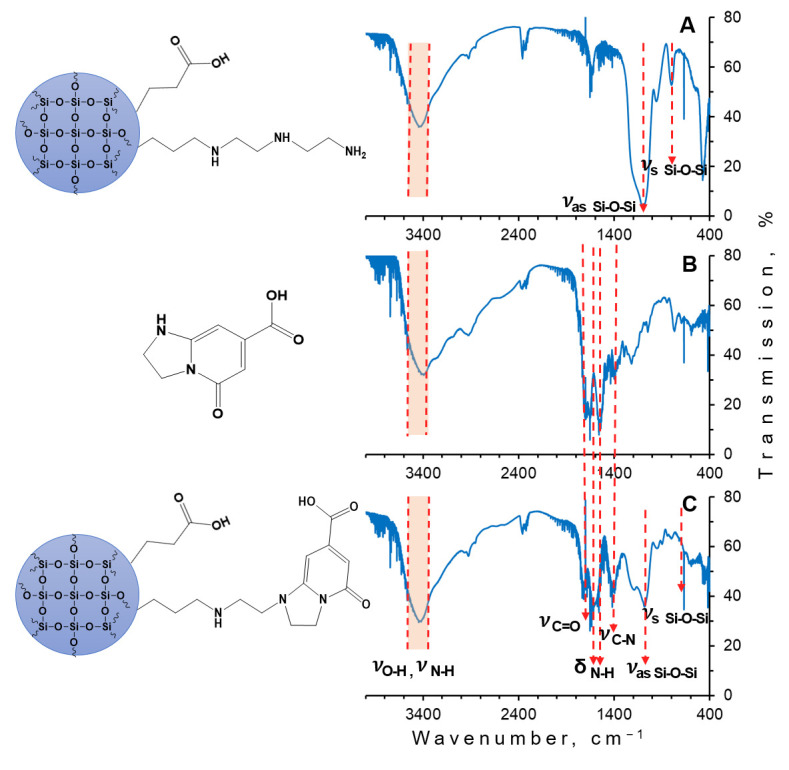
FTIR spectra of silica nanoparticles (**A**), IPCA molecular fluorophore (**B**), and luminescent composite nanoparticles (**C**).

FTIR spectra (Figure 7) were used to study the structures and identify the functional groups of synthesized silica nanoparticles after modifications (Figure 7A), IPCA molecular fluorophore (Figure 7B), and the composites after dialysis (Figure 7C). All the FTIR spectra demonstrated similar features. The intense bands at 1080–1120 cm^−1^ and 800 cm^−1^ corresponded to the asymmetric and symmetric stretching vibrations of Si–O–Si, respectively. Similar bonds were presented at the spectra of the luminescent composites before and after dialysis. The broad band at 3300–3600 cm^−1^ was responsible for the stretching vibrations of Si-OH groups with the presence of hydrogen bonds. Comparing the FTIR spectra of silica nanoparticles and the composites, the new bands at the area of 3300–3400 cm^−1^ correlated with the stretching vibrations of N-H groups, and the band at 1650 cm^−1^ assigned with bending vibrations of aliphatic amines were observed. The band corresponding with the asymmetric stretching vibrations of C=O group was observed at 1680 cm^−1^. This band indicates the incorporation of CA into the fluorophore structure. The characteristic band at 1400 cm^−1^ was assigned with the vibrations of C-N group. These bands can be evidence of the presence of the fluorophore on the silica matrix surface.

## 4. Conclusions

During the fifteen years of intensive research on CNSs, the problem associated with the synthesis of controlled morphology and functionality of CNSs has not been solved yet. This limits the fundamental understanding of their optical properties and reduces possibilities of their application. To achieve control over the properties of CNSs, in this work, we proposed a targeted one-stage hydrothermal synthesis of an organic fluorophore of the IPCA- type on the surface of a silica nanoparticles. This approach can be applied to the modification of other matrix. The application of the silica nanoparticles for the synthesis of luminescent composites had several important advantages such as uniform size and properties of nanostructures, the homogeneous distribution of fluorophore molecules within silica matrix, the presence of uniform carboxylic functional groups, relatively simple preparation process, and environmental safety. Thus, the application of a silica matrix makes it possible to eliminate the problems associated with the purification and separation of the luminescent product. In this work, the conditions for the synthesis and modification of the surface of the silica matrix were optimized. To avoid aggregation of the silica matrix during the modification by aminosilane DETAS, the stabilizing carboxysilane CEST was introduced. The colloidal stability of silica nanoparticles was observed at over 50% of CEST in the mixture. An increase in the amount of CEST led to decreases in the average size and PI as well as an increase in the negative ζ-potential. Spherical luminescent composites based on a modified silica matrix and CA with an overall size of ~26 nm were obtained through the simple hydrothermal method. The composites were formed due to the double cyclization process between the carboxylic groups of CA and amino groups of the modified silica matrix. To obtain the most luminescent and colloidal stable composite, a matrix with a 50:50 DETAS:CEST ratio was used. Hydrothermal treatment at 180 °C for 3 h was optimal for the synthesis of composites, since the highest luminescence was observed. After the purification by dialysis, the luminescent composites were characterized by a luminescence QY of 65 ± 4%.

Thus, the preparation of luminescent composites consisting of silica nanoparticles and organic fluorophore by hydrothermal approach makes it possible to achieve control over their morphology and functionality and eliminate the problems associated with their fractionation.

The proposed approach for the single-stage hydrothermal synthesis of the luminescent composite nanoparticles is universal since the surface of the silica matrix can be modified with a variety of functional groups to interact with luminescent components (including the formation of other fluorophores) for the specific research. High QY, colloidal stability, and monodispersity allow for applying these luminescent composites as prospective analogs of organic dyes and quantum dots in medical, biological, and analytical fields.

## Figures and Tables

**Table 1:** The hydrodynamic size, polydispersity index, and ζ-potential of modified by DETAS/CEST silica nanoparticles.

Surface Functionalization		Hydrodynamic Size, nm	Polydispersity Index	ζ-Potential, mV
DETAS, %	CEST, %			
50	50	165 ± 4	0.16 ± 0.02	−39 ± 3
40	60	108 ± 2	0.15 ± 0.01	−43 ± 3
20	80	96 ± 2	0.13 ± 0.01	−43 ± 4
0	100	66 ± 1	0.12 ± 0.01	−52 ± 4

**Table 2:** Morphological, electrokinetic, and QY values of obtained composites from CA and silica nanoparticles with different DETAS:CEST ratio.

DETAS:CEST ratio	20:80	40:60	50:50
**Average size, nm**	34 ± 7	38 ± 4	43 ± 2
**PI**	0.56 ± 0.04	0.31 ± 0.02	0.27 ± 0.04
**ζ-potential, mV**	−20 ± 2	−21 ± 3	−31 ± 2
**QY, %**	37 ± 3	43 ± 4	66 ± 2

## Data Availability

Not applicable.

## Supplementary Materials

The following supporting information can be downloaded at https://www.mdpi.com/article/10.3390/ma15238469/s1, Figure S1: Schematic illustration of the formation mechanism of IPCA molecular fluorophore from CA and EDA during hydrothermal synthesis [20]; Figure S2: ^1^H-NMR of IPCA organic fluorophore; Figure S3: Schematic illustration of the obtained composite structure (A); The ^1^H-NMR spectrum of final composite nanoparticles after dialysis (B); Figure S4: XRD pattern of the luminescent composite nanoparticles; Figure S5: TEM images of modified silica nanoparticles (A) and obtained luminescent composite nanoparticles (B).
