# Supplementary material for: A Facile Approach to the Hydrothermal Synthesis of Silica Nanoparticle/Carbon Nanostructure Luminescent Composites
Source: Materials (Basel). 2022 Nov 28;15(23):8469. doi: 10.3390/ma15238469 (PMC9737401; doi:10.3390/ma15238469)
Supplement: Supplementary file 1 [file materials-15-08469-s001.zip › materials-2009857-supplementary.pdf]

SUPPORTING INFORMATION

A Facile Approach to the Hydrothermal Synthesis of Silica Nanoparticle/Carbon Nanostructure Luminescent Composites

Yuliya A. Podkolodnaya *, Alina A. Kokorina and Irina Yu. Goryacheva

Department of Inorganic Chemistry, Chemical Institute, Saratov State University, Astrakhanskaya Street 83, 410012 Saratov, Russia;
alinaa.kokorina@gmail.com (A.A.K.); goryachevaiy@mail.ru (I.Y.G.)
* Correspondence: podkolodnaya00@mail.ru

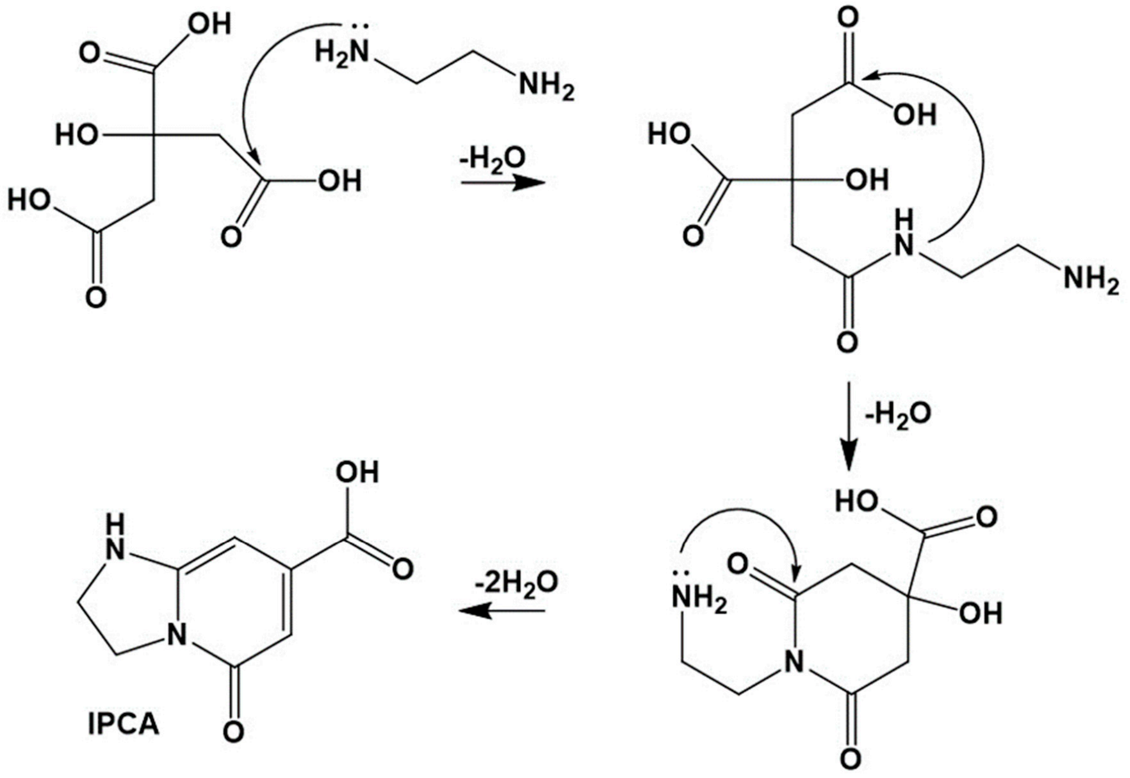

Figure S1. Schematic illustration of the formation mechanism of IPCA molecular fluorophore from CA and EDA during hydrothermal synthesis [20].

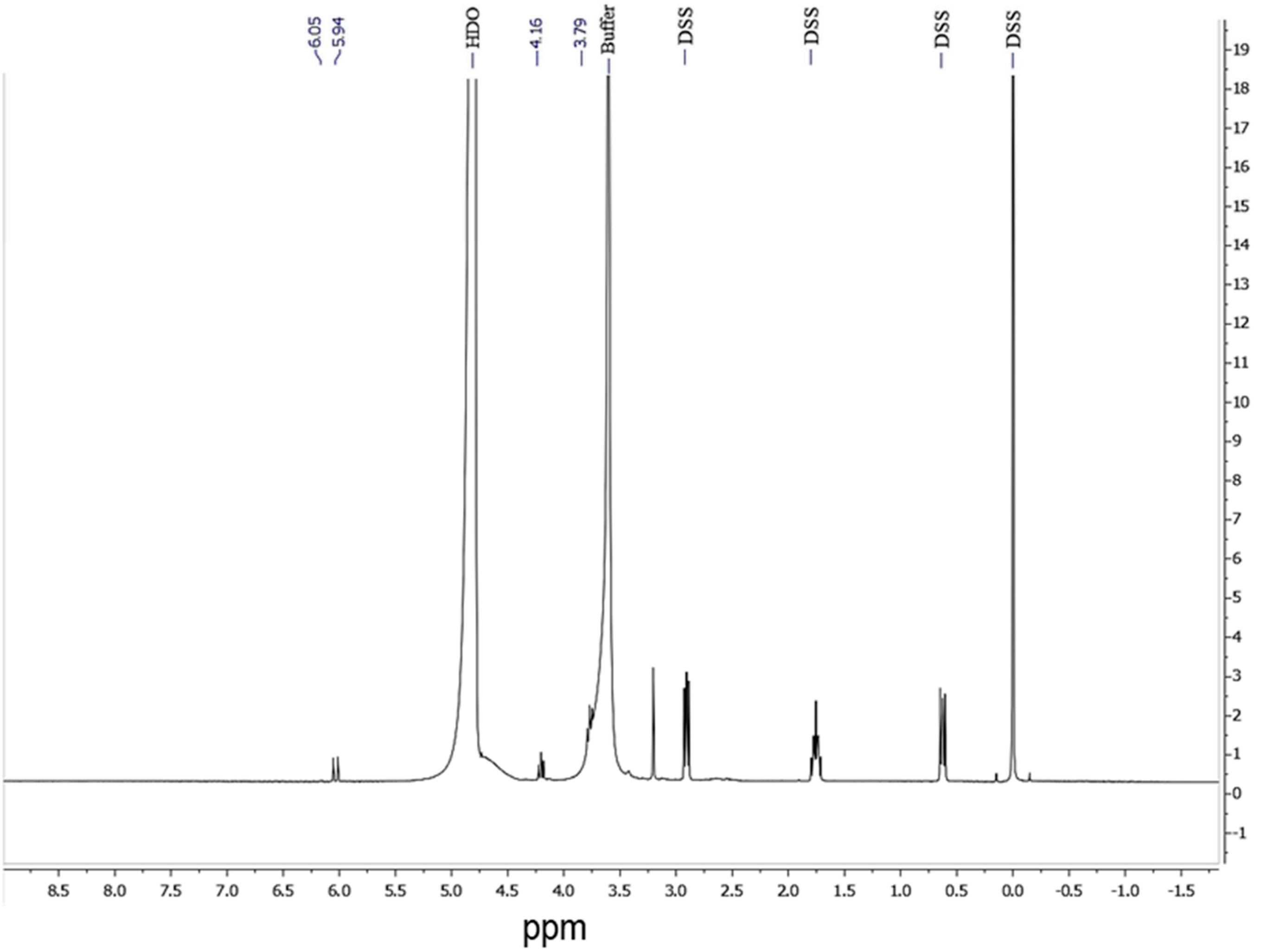

Figure S2. ^{1}H-NMR of IPCA organic fluorophore.

The ^{1}H-NMR spectrum of IPCA contains all the characteristic signals of aliphatic protons as triplets at 3.79 and 4.16 ppm, and the signal of protons in the pyridone ring at 5.94 and 6.05 ppm.

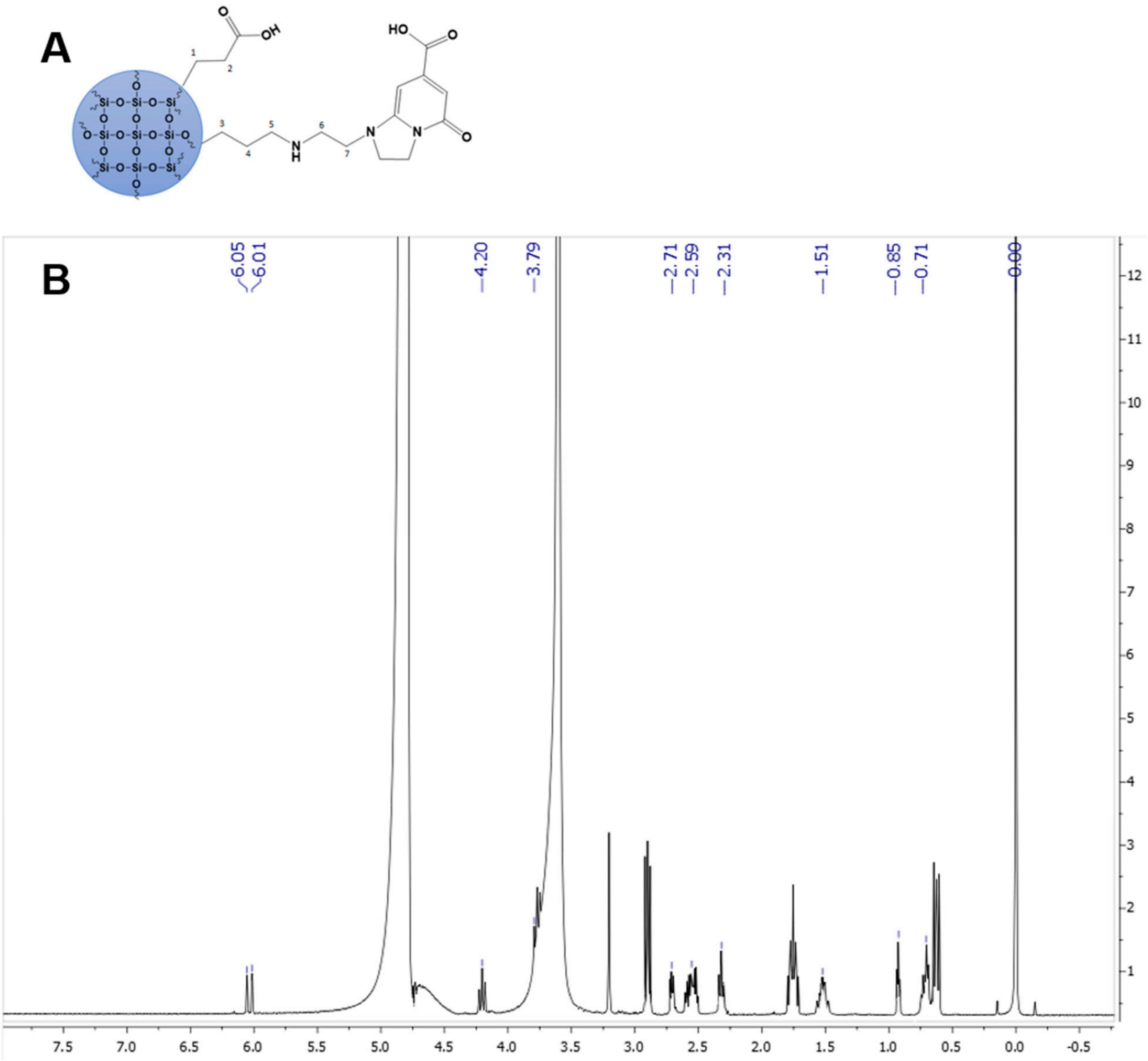

Figure S3. Schematic illustration of the obtained composite structure (A); The ^{1}H-NMR spectrum of final composite nanoparticles after dialysis (B).

The composite ^{1}H-NMR spectrum contains the signals corresponding to the IPCA structure and shows the signal of aliphatic protons of the matrix modificators. There are the signals of the methylene units of the acid modificators as a triplet at 2.31 ppm. (CH_2 group No. 1), and a signal at 0.85 ppm (methylene group No. 2). There are also multiples at 0.71 ppm. (No. 3), 1.15 ppm (No. 4), 2.59 ppm (No. 5,6), and 2.71 ppm. (No. 7) corresponding to the methylene units of the aminoalkyl fragments.

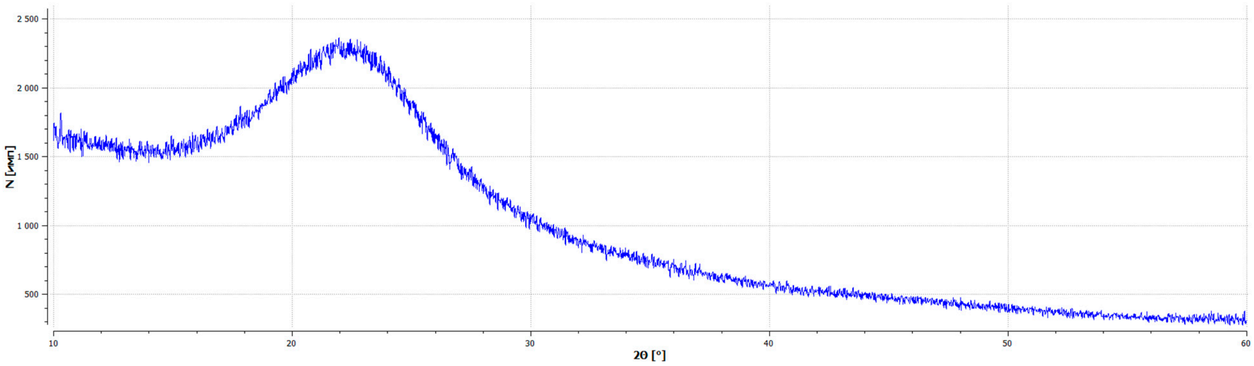

Figure S4. XRD pattern of the luminescent composite nanoparticles.

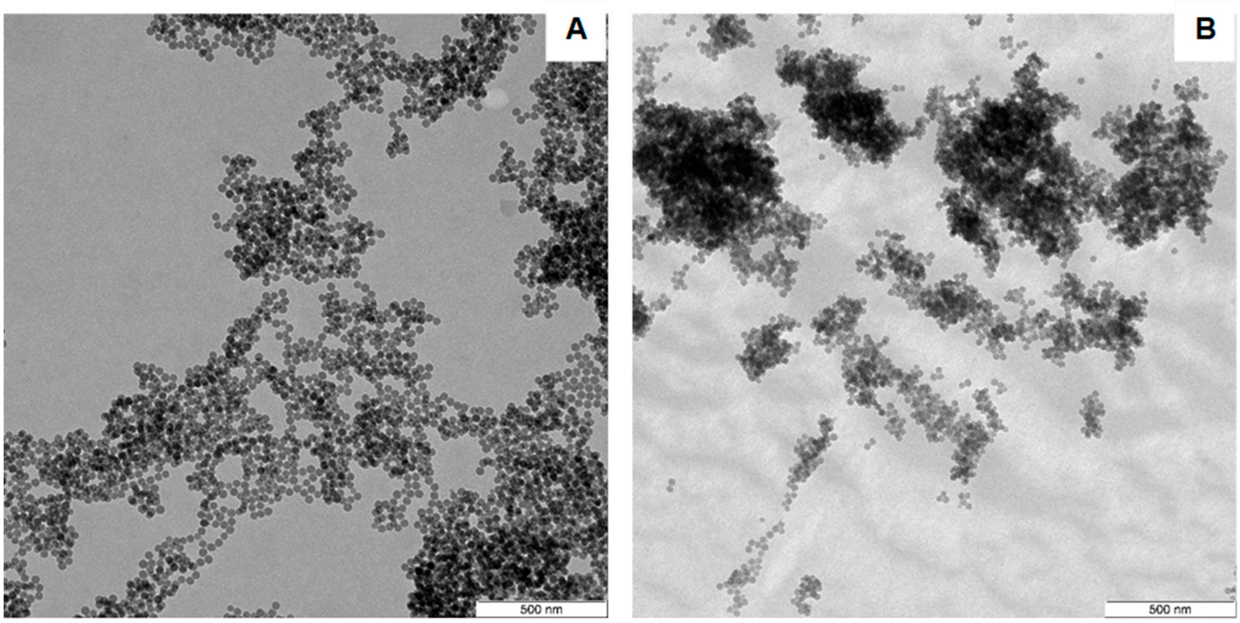

Figure S5. TEM images of modified silica nanoparticles (A) and obtained luminescent composite nanoparticles (B).
